# Lipid droplet - mitochondria coupling: A novel lipid metabolism regulatory hub in diabetic nephropathy

**DOI:** 10.3389/fendo.2022.1017387

**Published:** 2022-10-25

**Authors:** Ming Yang, Shilu Luo, Jinfei Yang, Wei Chen, Liyu He, Di Liu, Li Zhao, Xi Wang

**Affiliations:** ^1^ Department of Nutrition, Xiangya Hospital, Central South University, Changsha, China; ^2^ Department of Nephrology, The Second Xiangya Hospital of Central South University, Changsha, China; ^3^ Department of Reproduction and Genetics, The First Affiliated Hospital of Kunming Medical University, Kunming, China; ^4^ National Clinical Research Center for Geriatric Disorders, Xiangya Hospital, Central South University, Changsha, China

**Keywords:** peridroplet mitochondria, lipid droplets, diabetic nephropathy, mitochondria, β-oxidation

## Abstract

Diabetic nephropathy (DN) involves serious lipid metabolism disorder, and renal ectopic lipid deposition aggravates DN progression. However, the molecular mechanism of renal lipid deposition in DN remains unclear. Lipid droplets (LDs) are lipid pools in cells that change dynamically in response to the cellular energy needs. The LDs and mitochondria are connected through a part of the mitochondria known as the peridroplet mitochondria (PDM). In this review, we summarize the definition, detection methods, and function of the PDM. Finally, we discuss the research status of PDM in DN and the possibility of its use as a therapeutic target.

## 1 Introduction

Presently, the number of diabetes patients is increasing worldwide. As a systemic metabolic disease, diabetes may cause serious microvascular complications such as diabetic nephropathy (DN) (1). DN has gradually become the leading cause of end-stage renal disease (ESRD) in developed countries. However, specific drugs for DN are not available till date; therefore, DN pathogenesis should be explored urgently to develop new therapeutic drugs (2–4). Lipid metabolism disorder is a key factor in DN progression. Ectopic lipid deposition is aggravated in DN, which further promotes tubule cell inflammation and apoptosis and ultimately aggravates the pathological changes of DN (5–7). However, the mechanism of abnormal lipid deposition in DN remains unclear.

The cell is an organic entity and the organelles are not completely separated; in particular, organelles such as mitochondria-associated endoplasmic reticulum membranes (MAMs) are connected (8, 9). MAM integrity maintenance is essential for signal communication and cell homeostasis (10–12). Lipid droplets (LDs), as energy-storing organelles in cells, are also closely linked to the mitochondria. The mitochondria part closely connected to the LDs is also called peridroplet mitochondria (PDM) (13–15). The PDM plays a key role in maintaining lipid homeostasis and energy metabolism, and its abnormality can cause metabolic disorders (16, 17). In this review, we systematically summarized the progress of studies on PDM and discussed its potential role in DN.

## 2 LD structure

LD is a dynamic organelle, and this dynamic behavior reflects the overall cellular metabolic level (18–21). LD are also energy storing organelles that rapidly mobilize lipids to release fatty acids to provide cellular energy through β-oxidation. Moreover, LDs inhibit lipotoxicity caused by free fatty acids by isolating lipids (22–24). LDs are ubiquitous in cells and have a single phospholipid bilayer that insulates neutral lipids from the cytoplasm, protecting cells from the toxicity of free fatty acids (25). Furthermore, various proteins are embedded in the phospholipid bilayer to mediate different functions of the LDs (26–28). Approximately 200 proteins located on LDs have been identified (29), such as Rab GTPase (30, 31), PNPLA family (32, 33), and LXRα (34), with the development of biological techniques. These proteins play a key role in maintaining the stability of LD structure and timely response to nutritional status. There are two main mechanisms of LD decomposition: lipolysis (35, 36) and lipophagy (37–39). Lipolysis is the release of free fatty acids from triacylglycerol under the sequential action of adipose triglyceride lipase (ATGL), hormone-sensitive lipase (HSL), and monacylglycerol lipase (MAGL), whereas lipophagy is a form of selective autophagy in which lipid droplets are swallowed by the autophagosome membrane and fused with lysosomes, which are degraded by hydrolases (40). Several studies have indicated that LDs dynamically interact with other organelles, particularly the mitochondria, endoplasmic reticulum, endosomes, and peroxisomes, which is essential for maintaining cellular energy metabolism homeostasis.

## 3 β-Oxidation in mitochondria

Mitochondria are the center of cellular energy metabolism, where fatty acids can undergo β-oxidation to provide energy for cells. Fatty acid oxidation pathway can be divided into α-oxidation, β-oxidation and ω-oxidation, among which α-oxidation and ω-oxidation only exist in eukaryotes, while β-oxidation is the main fatty acid degradation pathway in eukaryotes or prokaryotes (41, 42). The fatty acids are activated by combining with coenzyme A (CoA) to form fatty acid acyl-CoA esters to participate in metabolic pathways. In the presence of ATP and Mg^2+^, the acyl portion of fatty acids is linked to the sulfur atoms in CoA through thioester bonds to form fatty acyl-CoA derivatives, pyrophosphate (PPi), and adenosine monophosphate (AMP) (43). Subsequently, the fatty acyl-CoA ester is converted to acyl-carnitine in the presence of l-carnitine, which enters the outer mitochondrial membrane. L-carnitine acyl-transferases subtypes, carnitine palmitoyltransferase-1 (CPT1) and carnitine palmitoyltransferase-2 (CPT2), have been detected in humans. Acyl-carnitine crosses the inner mitochondrial membrane under the action of carnitine acyl-carnitine translocase (CACT). Then, CPT2 regenerates the fatty acyl-CoA ester, which is successively dehydrogenated, hydrated, dehydrogenated, and thiolyzed to form acetyl-CoA by the fatty acid β-oxidase system in the mitochondrial matrix, to participate in energy generation (43).

## 4 What is the PDM?

In cells, mitochondria are the energy factories and LDs are energy pools. The latest studies have suggested connections between the mitochondria and LD, which were first observed in 1959 (44). With the development of biotechnology, several studies have revealed the link between LDs and mitochondria, and the LD-associated mitochondrial part is called PDM (45, 46). In fact, the biological functions, proteins, crest structure, and dynamics of PDM and cytoplasmic mitochondria are different (45). Recently, as the PDM has attracted attention, the quantifiable parameters and definition of PDM has also been proposed: a. Mitochondria in direct contact with LD that can be observed by electron microscopy; b. This part of the mitochondria is tightly linked to the LD, even after mechanical cell disruption or purification (45).

## 5 Molecular linkage mechanism and function of PDM

### 5.1 Proteins mediating PDM integrity

As dynamic organelle junctions, PDM integrity adjusts itself according to the cellular energy requirements, which is precisely regulated by certain proteins. Till date, some proteins involved in the coupling between LDs and mitochondria have been identified, and their loss destroys PDM integrity.

#### 5.1.1 SNAP23

SNAP23 belongs to the family of soluble N-ethylmaleimide-sensitive factor attachment protein receptor (SNARE) proteins that share a unique SNARE motif in the form of conserved sequences of 60–70 amino acids (47). The SNARE motifs mediate SNARE complexes formation and, thus, play a key role in intimal fusion in many cells. SNAP23 is also involved in maintaining PMD integrity. Lim et al. demonstrated that ADP-ribosylation factor (ARF)-related protein 1 (ARFRP1) recruits SNAP23 to a site close to LD, thereby promoting LD growth in hepatitis C virus (HCV) infected cells (48). This suggested that SNAP23 is essential for LD expansion. Furthermore, Sadh et al. reported that SNAP23 level was higher in LDs in the fasted mice liver than in the control mice liver, which is accompanied by increased LD-mitochondria interactions (49). Similarly, Strauss et al. showed that SNAP23 co-localizes mainly with the mitochondria in healthy trained lean human muscles (50). These studies strongly suggest that SNAP23 is closely related to LDs and mitochondria. Moreover, Jagerstrom et al. demonstrated a direct relationship between SNAP23 and PDM, further suggesting that SNAP23 downregulation reduces mitochondrial β-oxidation, which is accompanied by PDM integrity destruction (51).

#### 5.1.2 Perilipin 5 (PLIN5)

PLIN5 is a 463-residue protein that belongs to the perilipin protein family, which is expressed in highly oxidized tissues such as the heart and oxidized skeletal muscle. In high fat-fed PLIN5^−/−^ mice, the liver TAG content and expression of FA synthesis-related enzymes decreased (52). Moreover, PLIN5 plays an important role in regulating lipolysis by influencing the protein-protein interaction between ATGL and 1-acylglycerol-3-phosphate O-acyltransferase (ABHD5), an ATGL activator (53–55). Interestingly, PLIN5 is also closely related to mitochondrial homeostasis. Immunogold electron microscopy and western blots of isolated mitochondria have indicated that PLIN5 is also expressed in not only LDs but also the mitochondria (56). Moreover, PLIN5 expression correlates with mitochondrial respiration rate for lipid-derived substrates in rat muscle (56). Interestingly, fatty acid respiration did not increase in mitochondria isolated from PLIN5-overexpressed muscles, while lipid oxidation increased in the homogenate containing PLIN5-coated LDs (56). Similarly, Andersson et al. showed that PLIN5 deficiency significantly damages the oxidative capacity of mitochondria in cardiomyocytes, is accompanied by the changes in lipid acyl composition of mitochondrial membrane phospholipids, and significantly reduces mitochondrial membrane depolarization (57). Thus, PLIN5 is involved in fatty acid oxidation from LDs to mitochondria. Furthermore, LD accumulation, mitochondrial dysfunction, and apoptosis increased, while PLIN5 expression substantially decreased in palmitic acid-treated cardiomyocytes (58). Acetylcholine treatment significantly upregulated PLIN5 expression, improved LD lipolysis, and increased LD-mitochondrial connection integrity, whereas PLIN5 knockout destroyed the protective effect of acetylcholine (58). Similarly, PLIN5 mRNA and protein levels were notably upregulated in hydrogen peroxide or lipopolysaccharide (LPS)-treated HepG2 cells, which increased LD-mitochondrial contact and downregulated intracellular ROS levels (59). Further studies determined that PLIN5 is localized at the LD-mitochondrial junction in human skeletal muscles through stimulated emission depletion (STED) microscopy and correlative light-electron microscopy (CLEM) performed (60). Although PLIN5 is involved in LD-mitochondria connection formation, the specific molecular mechanism is still unclear. An unidentified mitochondrial outer membrane protein may be involved in this process, which should be verified in further studies.

**Figure 1:**
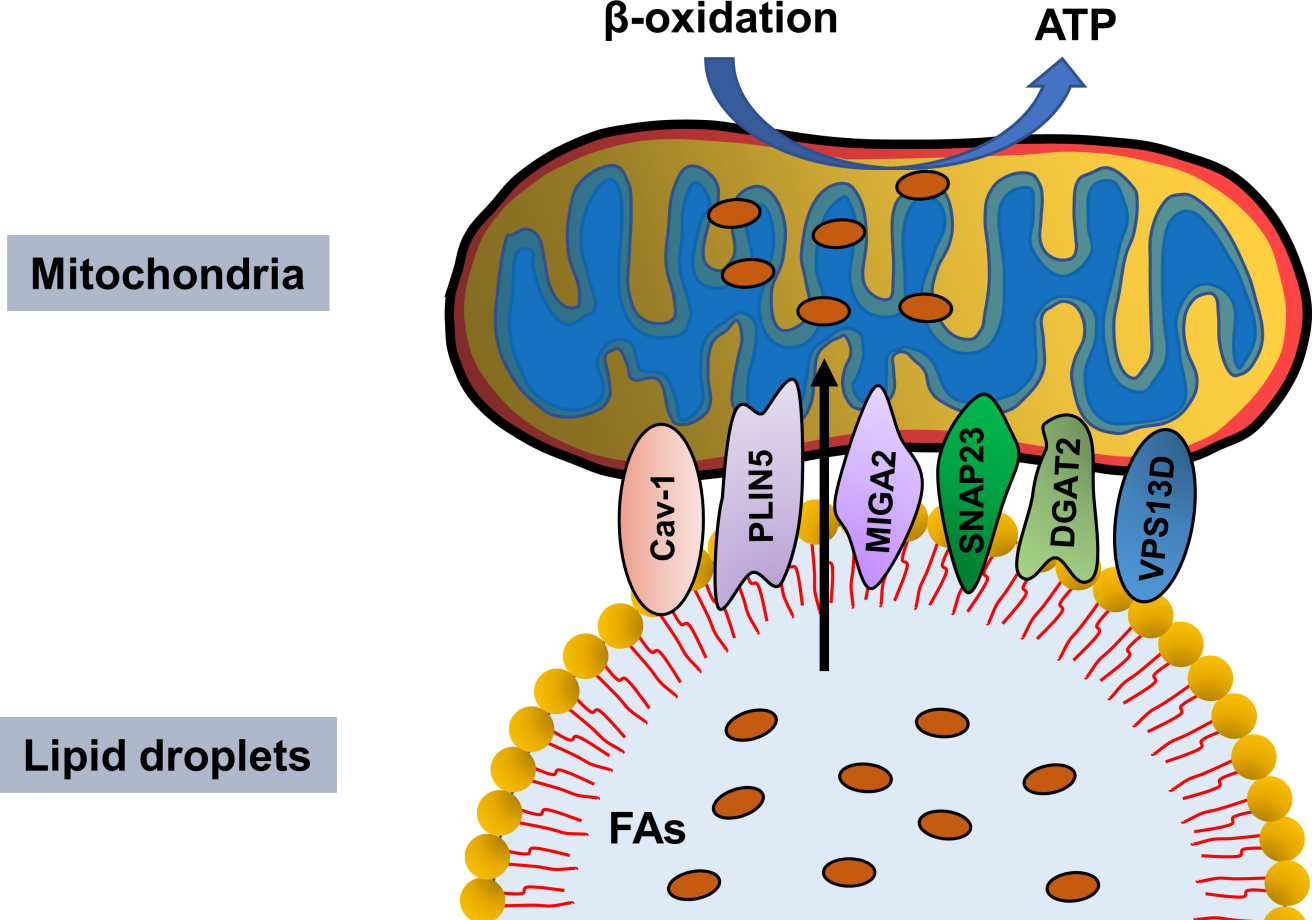
Proteins mediating lipid droplet and mitochondria integrity. Cav-1, PLIN5, MIGA2, SNAP23, DGAT2 and VPS13D maintain lipid droplet–mitochondria connection, thus facilitating fatty acids transfer in from lipid droplet to mitochondria for β-oxidation and ATP generation.

Some studies revealed that in addition to SNAP23 and PLIN5, other proteins mediate PDM integrity. Caveolin-1 (Cav-1), a multifunctional membrane protein, is the main component of caveolae and plays an important role in regulating endocytosis, stress response, and signal transduction (61). Meanwhile, it is also a LD envelope protein involved in lipid metabolism and LD formation. Kuo et al. showed impaired LD formation in Cav-1-deficient endothelial cells (62). Interestingly, its expression is also closely related to mitochondrial function. Wang et al. demonstrated that hippocampal overexpression of neuron-targeted Cav-1 significantly reduces mitochondrial damage and enhances mitochondrial respiration (Wang et al., 2021). Similarly, Cav-1 deficiency notably reduced mitochondrial respiration, decreased oxidative phosphorylated complex I activity, and downregulated the NAD^+^/NADH ratio, ultimately inducing premature aging (63). In addition, the high-quality electron micrographs of intact LDs and other adipocyte components by high pressure and rapid tissue freezing indicated LD-mitochondria connection, while the absence of Cav-1 resulted in a significant and almost complete absence of mitochondria in contact with LDs (64). Moreover, acyl-CoA: diacylglycerol acyltransferase 2 (DGAT2), a key enzyme that catalyzes the synthesis of triacylglycerol, is primarily located in the endoplasmic reticulum (ER) under basic conditions, but using oleic acid promotes TG synthesis, which transfers DGAT2 to the LD surface, where it co-locates with the mitochondria (65). Mechanistically, DGAT2 interacts with the mitochondria through the 67 N-terminal amino acids of DGAT2, which could guide a red fluorescent protein to the mitochondria (65). Furthermore, mitoguardin-2 (MIGA2) (66), an outer mitochondrial membrane protein, and VPS13D are also involved in LD-mitochondria coupling (**Figure 1**). Although the structural proteins of mitochondria and LDs have been partially revealed, further studies should focus on the proteins involved in PDM integrity maintenance and the molecular mechanisms involved.

### 5.2 Function of PDM

Currently, the function of PDM is mainly focused on lipid metabolism. In cells, LDs are the storage organelles and mitochondria are the energy factories. Thus, as a link between them, PDM regulation is indispensable in dealing with cellular energy disturbances. On the one hand, PDM directly connect LDs with the mitochondria, which can efficiently promote fatty acids transfer into the mitochondria for oxidation, thus providing cells with sufficient energy to resist external stimuli; on the other hand, it also confines free fatty acids to a small range, thereby reducing the toxicity of free fatty acids to cells. Rambold et al. demonstrated that labeled fatty acids are stored in the LDs of nutrient-rich cells, while fatty acids are transferred from the LDs in starving cells mitochondria for oxidation. Interestingly, fatty acids are not released from free cytoplasmic pools, but from LD sources and this process required the LDs to be close to the mitochondria (67). These findings suggest that increased PDM integrity significantly promotes fatty acid translocation from LDs to mitochondria for energy production. In addition to fatty acid oxidation, PDM is also involved in LD expansion through providing ATP for TAG synthesis. Benador et al. showed that the purified PDM showed stronger pyruvate oxidation, electron transfer, and ATP synthesis than cytoplasmic mitochondria; moreover, the PLIN5-induced LD-mitochondrial integrity upregulated the ATP-synthase-dependent triacylglyceride synthesis (45). This seemingly contradictory result may be because PDM plays different roles in different nutritional states. In nutrient-rich cells, PDM could mobilize the mitochondria to supply ATP to speed up LDs synthesis; When exposed to external stress, PDM ensures rapid fatty acid transfer from LDs to mitochondria for oxidative energy production.

## 6 PDM detection methods

Transmission electron microscopy (TEM) is the most direct detection method of PDM, a sub-organelle structure (45, 66, 68, 69). The high resolution of TEM allows the direct observation of LD and mitochondria connection. However, quantifying PDM integrity is difficult and requires instrument precision. In addition, double staining of living cells is another method of detecting PDM by direct observation. PDM (colocalization area) can be observed under confocal microscopy by co-staining living cells with LD markers (BODIPY) and mitochondrial dye (Mito-tracker) (45). The advantage of this method is that the interaction between LDs and mitochondria can be directly observed, but PDM function and protein expression cannot be studied. The optimal method for functional studies is to extract PDM, and Benador et al. have developed a method to isolate PDM according to degree of PDM attachment to LD (70). This method allowed the observation of differences in PDM protein expression under different nutritional or disease states, but the purity of the extracted PDM is related to many factors. However, further work is needed to optimize this separation scheme to accommodate organizational differences. In addition to these methods, *in situ* proximity ligation assay (PLA) may also be a potential way to detect PDM and is an effective method to detect the connection between ER and mitochondria using marker proteins (71).

## 7 Potential role of PDM in DN

DN, as a metabolic disease, is often accompanied by lipid metabolism disorder and several studies have revealed that lipid metabolism disorder aggravates DN progression. Edelstein et al. demonstrated increased lipid deposition and enlarged intracellular LDs in DN patients undergoing renal biopsy than those in the control (72). Furthermore, the increased lipid deposition in DN is associated with the expression of lipid metabolism disorder-associated genes. In DN state, the expression of key proteins in the fatty acid β-oxidation pathway (PPAR-α, carnitine palmitoyltransferase 1, acyl-CoA oxidase, and L-FABP) and those mediating cholesterol efflux (ABCA1, ABCG1, and apoE) notably decreased, while the expression of lipid uptake receptors, such as LDL, oxidized LDL, and acetylated LDL receptors, are significantly upregulated (72). Moreover, the degree of lipid disturbance was closely related to renal inflammation and glomerular filtration rate (72). Similarly, we have also showed that renal lipid deposition aggravates with the progression of pathological stages of DN and is associated with renal tubular interstitial damage (Yang et al., 2021). However, liraglutide, a novel hypoglycemic agent, improves renal outcomes in type 2 diabetes patients by inhibiting lipid synthesis and promoting lipid lipolysis in DN rats (73). These findings strongly suggest that lipid metabolism disorders are a risk factor for DN progression, and that DN patients may benefit from ameliorating lipid metabolism disorders.

**Figure 2:**
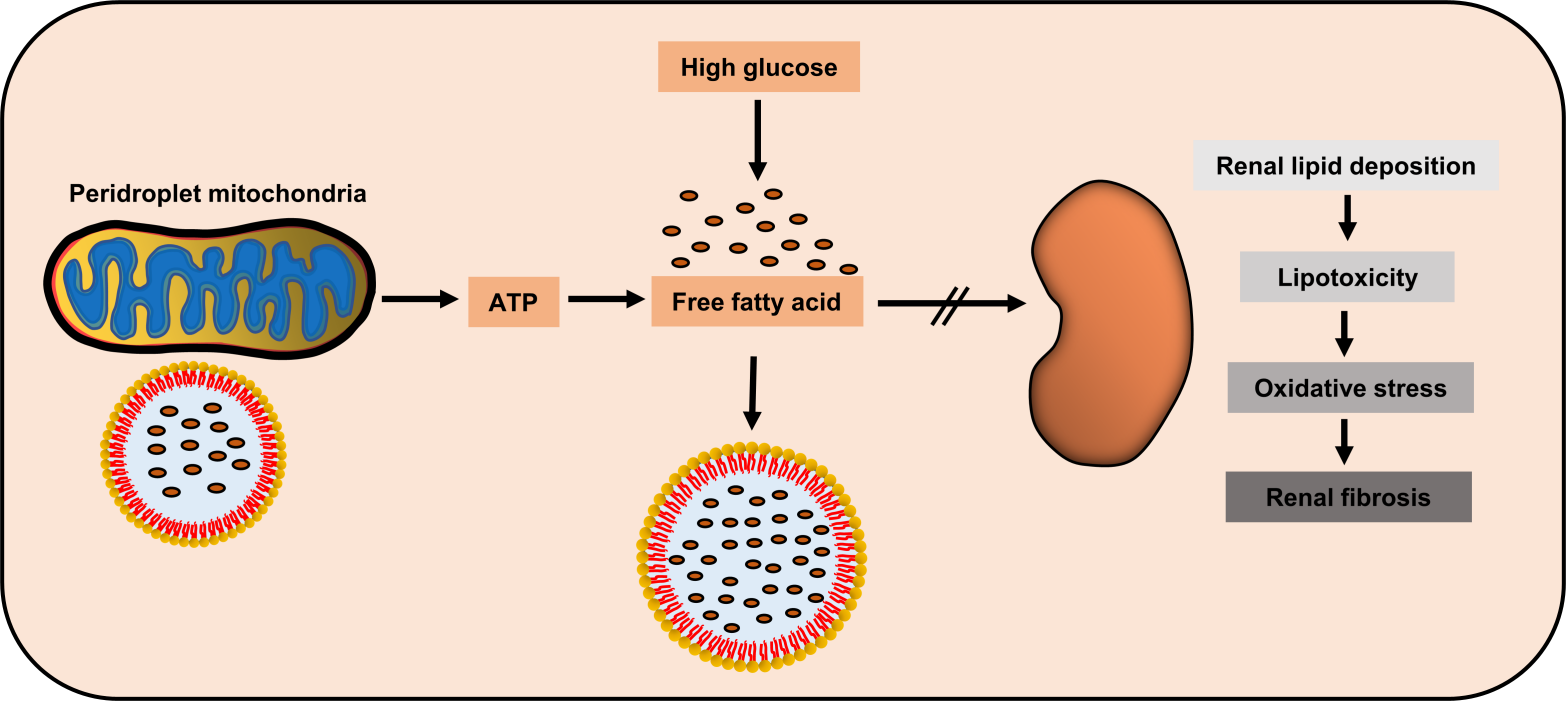
Potential renal protection of peridroplet mitochondria (PDM) in diabetic nephropathy. High glucose could induce the kidney to produce high amount of free fatty acids, while PDM generate ATP to promote lipotoxic fatty acids transfer into lipid droplets to be isolated, to protect kidney cells.

As an organ with high oxygen consumption, adequate energy supply helps maintain the steady state of kidney function. Renal proximal tubule cells utilize non-esterified fatty acids to maximize ATP production through β-oxidation (74). During DN, increased ROS and hyperglycemia changes the electron transport chain, thus reducing ATP production and increasing apoptosis (75). Moreover, Rossi et al. compared the renal proteomic data and blood metabolic profiles of Timp3-knockout and control mice, showing abnormal fatty acid β-oxidation, which is further aggravated after STZ induced DN (76). Similarly, high glucose treatment induced fatty acid deposition and downregulated β-oxidation rate in cultured human proximal tubule cells, thus changing in the phenotype of epithelial-to-mesenchymal transition (EMT); moreover, acetyl-CoA carboxylase 2 (ACC2) siRNA treatment accelerated β-oxidation rate, thus alleviating the adverse effects of high glucose (77). In fact, the impaired lipids of mitochondrial β-oxidation could be used as DN progression markers (78). These studies suggest abnormal fatty acid β-oxidation in the kidney during DN. Furthermore, accelerated fatty acids β-oxidation could ameliorate diabetic kidney injury (74, 79). Fenofibrate, a commonly used drug for DN, plays a protective role by promoting fatty acid β-oxidation (80, 81) (**Figure 2**).

Although few studies have focused on PDM in diseases and the relationship between PDM and DN is unclear, PDM may have some potential link with DN. On one hand, PDM can promote fatty acids β-oxidation, which protects against DN. On the other hand, PDM can also promote LD synthesis, which restricts the free fatty acids in the cytoplasm to LDs, thus, greatly reducing the lipotoxicity. This needs to be verified by further studies, but as an emerging research hotspot, the role of PDM in metabolic diseases, particularly diabetic kidney diseases, deserves further discussion.

## 8 Conclusion

LDs and mitochondria are important energy storage and productivity factories in cells and the interaction between LDs and mitochondria are involved in maintaining of cell energy metabolism homeostasis. Very few studies have focused on their interaction till date. DN is a metabolic disorder and lipotoxicity and mitochondrial energy imbalance play a key role in its progression. Interestingly, PDM is critical in reducing lipotoxicity and accelerating energy generation. However, no studies have focused on the interaction between PDM and DN. In this review, we have summarized the definition, detection, and function of PDM and explores the potential protective role of PDM in DN. However, many questions remain unsolved. In addition to lipid metabolism and LD synthesis, does PDM maintain intracellular mitochondrial homeostasis? Besides lipid deposition, does PDM integrity change in the kidney tissue of DN patients? What are the molecular mechanisms mediating PDM integrity destruction? Are there compounds that specifically regulate PDM integrity? Although many questions to be addressed in future research, PDM provides a new perspective for us to further understand and prevent DN progression.

## Author contributions

MY and XW wrote the manuscript, SL, JY, WC, LH, DL, and LZ edited the manuscript. All authors read and approved the final manuscript.

## Funding

This work was supported by the National Natural Science Foundation of China (81900069, 82000697).

## Conflict of interest

The authors declare that the research was conducted in the absence of any commercial or financial relationships that could be construed as a potential conflict of interest.

## Publisher’s note

All claims expressed in this article are solely those of the authors and do not necessarily represent those of their affiliated organizations, or those of the publisher, the editors and the reviewers. Any product that may be evaluated in this article, or claim that may be made by its manufacturer, is not guaranteed or endorsed by the publisher.
